# Atrioventricular Delay Optimization in His‐Optimized Cardiac Resynchronization Therapy: A Case Report

**DOI:** 10.1002/ccr3.71886

**Published:** 2026-01-18

**Authors:** Minh Nguyen Quang, Dung Kieu Ngoc, Son Nguyen Khac Le, Phuong Tran Le Uyen, Thuc Nguyen Tri

**Affiliations:** ^1^ Arrhythmology Department Cho Ray Hospital Ho Chi Minh City Vietnam; ^2^ Ministry of Health Vietnam

**Keywords:** AV delay optimization, cardiac resynchronization therapy, conduction system pacing, His bundle pacing, His‐optimized CRT

## Abstract

His bundle pacing (HBP) has recently emerged as a physiologic pacing strategy that preserves ventricular synchrony and may improve outcomes in patients with heart failure and conduction abnormalities. Optimization of the atrioventricular (AV) delay plays a pivotal role in ensuring hemodynamic efficiency in patients with conduction system pacing. We report a 41‐year‐old male with dilated cardiomyopathy and severe heart failure (NYHA class IV, EF 16%), who was initially implanted with a cardiac resynchronization therapy (CRT) device. Despite multiple attempts to reposition the right and left ventricular leads, QRS duration during biventricular pacing widened from 140 to 185 ms. The right ventricular lead was subsequently repositioned to the His bundle (His‐optimized CRT, HOT‐CRT). Following AV delay optimization, the QRS duration narrowed to 120 ms, improving hemodynamic function. At 15‐month follow‐up, the patient remained clinically stable, able to perform moderate physical activity, with an improved EF of 25% and no recurrence of ventricular tachycardia. HOT‐CRT combines the physiologic advantage of His bundle pacing with optimized AV conduction timing, which is particularly valuable in patients with prolonged PR interval and nonresponse to conventional CRT. AV delay optimization ensures proper atrioventricular synchrony, enhances left ventricular filling, and contributes to improved cardiac output. This case highlights the significance of individualized AV delay adjustment in conduction system pacing to maximize therapeutic outcomes. Optimization of AV delay is essential for maximizing the benefits of conduction system pacing, especially in HOT‐CRT recipients. Attention to QRS morphology and duration during device implantation can help identify patients who will benefit most from individualized AV programming.

## Key Clinical Message

HOT‐CRT with individualized AV delay optimization can rescue patients who fail conventional CRT, particularly those with prolonged PR interval and intraventricular conduction delay. Dramatic QRS narrowing and hemodynamic improvement highlight the value of tailored conduction–system pacing strategies.

## 1 Introduction

Heart failure is commonly associated with electrical dyssynchrony, including intraventricular conduction delay and first‐degree AV block, both of which impair ventricular filling and worsen outcomes [1, 2]. Conventional right ventricular pacing may exacerbate dyssynchrony and can precipitate pacing‐induced cardiomyopathy when the ventricular pacing burden is high [3, 4].

To preserve physiological ventricular activation, conduction‐system pacing strategies—His bundle pacing (HBP) and left bundle branch pacing (LBBP)—have emerged as superior alternatives to right ventricular or even biventricular pacing [5, 6]. In patients with prolonged PR interval or suboptimal resynchronization after CRT, AV delay optimization is essential; a shorter programmed AV delay improves diastolic filling and hemodynamics [7, 8].

This case is unique because it demonstrates the use of His‐optimized CRT (HOT‐CRT) as a rescue strategy after failed conventional CRT in a patient with cardiogenic shock, dilated cardiomyopathy, prolonged PR interval, and intraventricular conduction delay. The dramatic QRS narrowing (185 → 120 ms) after AV delay optimization underscores the importance of individualized programming.

## 2 Case History/Examination

A 41‐year‐old man presented with acute dyspnea and hypotension (78/50 mmHg), cold extremities, and elevated lactate (3.2 mmol/L), fulfilling the criteria for cardiogenic shock. He had no prior ischemic disease; coronary angiography revealed normal coronaries, suggesting nonischemic dilated cardiomyopathy. Laboratory tests showed BNP 2350 pg/mL, mildly elevated high‐sensitivity troponin‐T (48 ng/L), and normal electrolytes.

**FIGURE 1:**
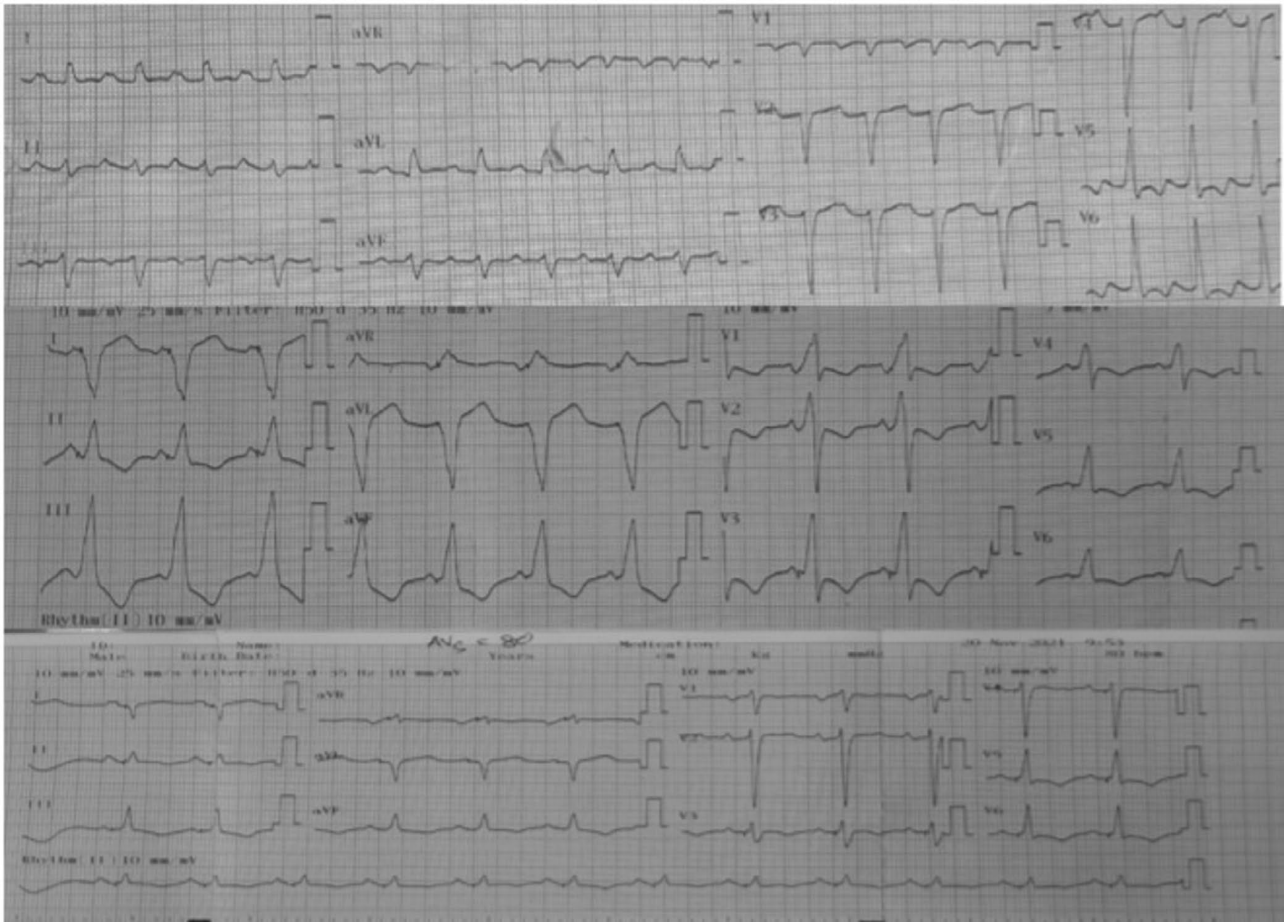
ECG before CRT, during CRT pacing, and after HOT‐CRT.

Baseline ECG demonstrated first‐degree AV block (PR 240 ms) and intraventricular conduction delay with QRS 140 ms (Figure 1). Echocardiography revealed a markedly dilated LV (LVEDD 75 mm) with an LVEF of 16%. Because of severe systolic dysfunction, anticipated high ventricular pacing burden due to PR prolongation, and documented nonsustained ventricular tachycardia, he met criteria for CRT‐D implantation.

## 3 Differential Diagnosis, Investigations, and Treatment

The differential diagnosis for cardiogenic shock included acute myocarditis, tachycardia‐induced cardiomyopathy, and advanced nonischemic dilated cardiomyopathy. Coronary artery disease, structural abnormalities, thyroid dysfunction, and reversible metabolic causes were excluded through appropriate testing.

CRT‐D implantation was performed. Despite multiple repositioning attempts of the right and left ventricular leads, biventricular pacing paradoxically widened the QRS duration from 140 to 185 ms, indicating failed resynchronization. Given the intraventricular conduction delay and prolonged PR interval, the right ventricular lead was repositioned to the His bundle, achieving nonselective His capture and creating a His‐optimized CRT (HOT‐CRT) configuration.

AV delay optimization was performed immediately after confirming stable His capture using combined ECG‐guided and hemodynamic‐guided assessment.

**FIGURE 2:**
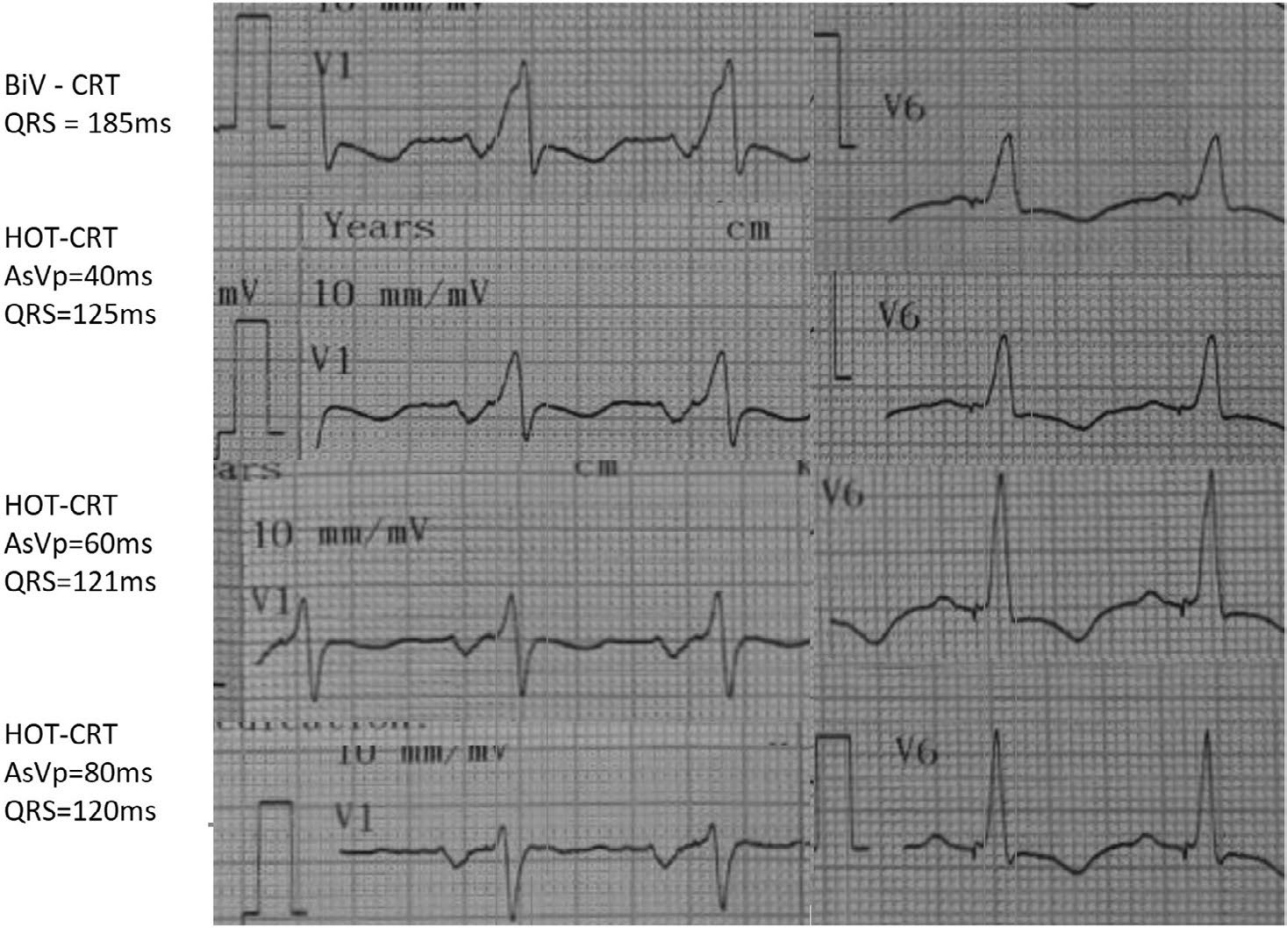
Post‐HOT‐CRT ECG after AV‐delay optimization showing QRS narrowing to 120 ms.

- Intrinsic PR interval: 240 ms
- Initial programmed AV delay: 200 ms (suboptimal with fusion and incomplete resynchronization)
- Method: stepwise AV delay reduction in 20‐ms increments while evaluating mitral inflow pattern, LVOT VTI, systolic blood pressure, and QRS morphology
- Optimal AV delay: 140 ms, resulting in:
  ○ complete separation of E/A waves;
  ○ LVOT VTI improvement (+18%);
  ○ systolic BP increase from 82 → 96 mmHg;
  ○ QRS narrowing to 120 ms (Figure 2)

This setting was programmed as the final therapeutic AV delay.

## 4 Conclusion and Results (Outcome and Follow‐Up)

The patient showed rapid hemodynamic improvement after AV delay optimization and was discharged on guideline‐directed medical therapy. At 15‐month follow‐up, the patient remained clinically stable (NYHA II), with an improved LVEF of 25% and no recurrent ventricular tachycardia.

## 5 Discussion

This case highlights the importance of recognizing early CRT nonresponse and considering conduction‐system pacing as a rescue strategy. Up to one‐third of patients fail to respond to CRT due to suboptimal LV lead position, scar burden, intraventricular conduction delay, or marked PR prolongation causing atrioventricular dyssynchrony [3, 4].

In this patient, CRT failure was likely driven by:

1. intraventricular conduction delay without classic LBBB morphology,
2. prolonged PR interval (240 ms) producing significant AV dyssynchrony, and
3. inability to achieve LV–RV synchrony, as evidenced by QRS widening to 185 ms under biventricular pacing.

His bundle pacing restores near‐physiologic activation of the His‐Purkinje system and may overcome limitations of conventional CRT in patients with intraventricular conduction delay [5, 6]. However, when intrinsic AV conduction is prolonged, individualized AV delay optimization becomes critical to achieving effective resynchronization.

Prior studies have demonstrated the hemodynamic benefits of tailored AV delay programming: Kosmala et al. reported improvements in LVEF and LVOT VTI with individualized optimization [8], while Salden et al. showed significant increases in cardiac output in patients with AV dyssynchrony [7].

In our case, HOT‐CRT combined with AV delay optimization produced marked QRS narrowing (185 → 120 ms), immediate hemodynamic improvement, and sustained clinical benefit—findings consistent with emerging evidence and the BRAVO trial [9].

This case emphasizes that HOT‐CRT should be considered when conventional CRT fails, and that AV delay optimization is pivotal for maximizing response in patients with prolonged PR interval and intraventricular conduction delay.

## Author Contributions

**Minh Nguyen Quang:** conceptualization, supervision, writing – original draft. **Dung Kieu Ngoc:** data curation, investigation. **Son Nguyen Khac Le:** methodology, validation, writing – review and editing. **Phuong Tran Le Uyen:** supervision, visualization. **Thuc Nguyen Tri:** formal analysis, writing – review and editing.

## Funding

The authors have nothing to report.

## Consent

Written informed consent was obtained from the patient for publication of this case and accompanying images.

## Conflicts of Interest

The authors declare no conflicts of interest.

## Data Availability

All data underlying this case report are included within the article. Additional clinical information is available from the corresponding author upon reasonable request, in accordance with patient privacy regulations.

## References

[1] S. Cheng , M. J. Keyes , M. G. Larson , et al., “Long‐Term Outcomes in Individuals With Prolonged PR Interval or First‐Degree Atrioventricular Block,” Journal of the American Medical Association 301, no. 24 (2009): 2571–2577, 10.1001/jama.2009.888. 19549974 PMC2765917

[2] M. Glikson , J. C. Nielsen , M. B. Kronborg , et al., “ESC Guidelines on Cardiac Pacing and Cardiac Resynchronization Therapy,” European Heart Journal 42, no. 35 (2021): 3427–3520, 10.1093/eurheartj/ehab364. 34455430

[3] M. N. Faddis , “Treatment of Pacing‐Induced Cardiomyopathy With Cardiac Resynchronization Therapy,” JACC. Clinical Electrophysiology 4, no. 2 (2018): 178–180, 10.1016/j.jacep.2017.11.012. 29749934

[4] S. Khurshid , E. Obeng‐Gyimah , G. E. Supple , et al., “Reversal of Pacing‐Induced Cardiomyopathy Following Cardiac Resynchronization Therapy,” JACC. Clinical Electrophysiology 4, no. 2 (2018): 168–177, 10.1016/j.jacep.2017.10.002. 29749933

[5] S. K. Padala , J.‐A. Cabrera , and K. A. Ellenbogen , “Anatomy of the Cardiac Conduction System,” Pacing and Clinical Electrophysiology 44, no. 1 (2021): 15–25, 10.1111/pace.14107. 33118629

[6] P. S. Sharma and P. Vijayaraman , “Conduction System Pacing for Cardiac Resynchronisation,” Arrhythmia & Electrophysiology Review 10, no. 1 (2021): 51–58, 10.15420/aer.2020.45. 33936744 PMC8076975

[7] F. C. W. M. Salden , P. R. Huntjens , R. Schreurs , et al., “Pacing Therapy for Atrioventricular Dromotropathy: A Combined Computational‐Experimental‐Clinical Study,” Europace 24, no. 5 (2022): 784–795, 10.1093/europace/euab248. 34718532 PMC9071072

[8] W. Kosmala and T. H. Marwick , “Meta‐Analysis of Effects of Optimization of Cardiac Resynchronization Therapy on Left Ventricular Function, Exercise Capacity, and Quality of Life in Patients With Heart Failure,” American Journal of Cardiology 113, no. 6 (2014): 988–994, 10.1016/j.amjcard.2013.12.006. 24461769

[9] Z. I. Whinnett , S. M. A. Sohaib , M. Mason , et al., “Multicenter Randomized Controlled Crossover Trial Comparing Hemodynamic Optimization Against Echocardiographic Optimization of AV and VV Delay of Cardiac Resynchronization Therapy: The BRAVO Trial,” JACC: Cardiovascular Imaging 12, no. 8 Pt 1 (2019): 1407–1416, 10.1016/j.jcmg.2018.02.014. 29778861 PMC6682561

